# Hemangioma of the male breast: Presentation after thorn injury

**DOI:** 10.1259/bjrcr.20200187

**Published:** 2021-01-13

**Authors:** Ali Imran Alwani, Mary R. Schwartz, Barrett C. Riddle, Richard Caplan, Lewis L. Ware

**Affiliations:** 1 Texas A&M College of Medicine, Bryan, Texas, United States; 2 Department of Pathology and Genomic Medicine, Houston Methodist Hospital, Houston, Texas, United States; 3 Department of Radiology, Houston Methodist Hospital, Houston, Texas, United States; 4 Department of Surgery, Houston Methodist Hospital, Houston, Texas, United States; 5 Houston Methodist Breast Care Center, Houston Methodist Hospital, Houston, Texas, United States

## Abstract

Hemangiomas of the breast are uncommon and, in males, almost always present as a palpable breast mass. Here, we report the case of a male patient who was diagnosed with a breast hemangioma following an incidental injury to his breast, which triggered symptoms that prompted clinical work-up. As this diagnosis likely would have otherwise not been made, it follows that benign breast masses in males may be underreported and underdiagnosed.

## Introduction

Hemangiomas of the breast are rare in males, with, to the best of our knowledge, only 16 previously reported cases.^1–5^ Given the rarity of this male breast lesion, exact prevalence is not known, with 1 study reporting 12 total lesions from a specimen sample of 1362 (prevalence of 0.9%), of which only 1 was from a male.^6^ Most breast tumors, including hemangiomas, and other breast lesions in males present as palpable masses.^7^ Here, we report the case of a male patient who did not initially have a palpable breast mass, but following a thorn stick, developed clinical symptoms and eventually was diagnosed with a hemangioma of the breast.

## Clinical presentation

A 64-year-old Caucasian gentleman, with a history of coronary artery disease status post coronary artery bypass-grafting, presented with an 8-week complaint of a palpable right breast mass. He recalled being stuck by a 4-inch thorn in the right breast approximately 3 months earlier, soon after which he developed localized pruritus and irritation. These symptoms prompted him to see his primary care physician, who ordered a diagnostic breast mammogram.

## Differential diagnosis

The differential diagnosis of a palpable mass in a male patient includes, but is not limited to, gynecomastia, pseudogynecomastia such as lipomastia, hemangioma, angiosarcoma, lymphangioma, fat necrosis, pseudoangiomatous stromal hyperplasia, and reactive lymphadenopathy.^7^

## Investigation/imaging/pathology findings

**Figure 1.**
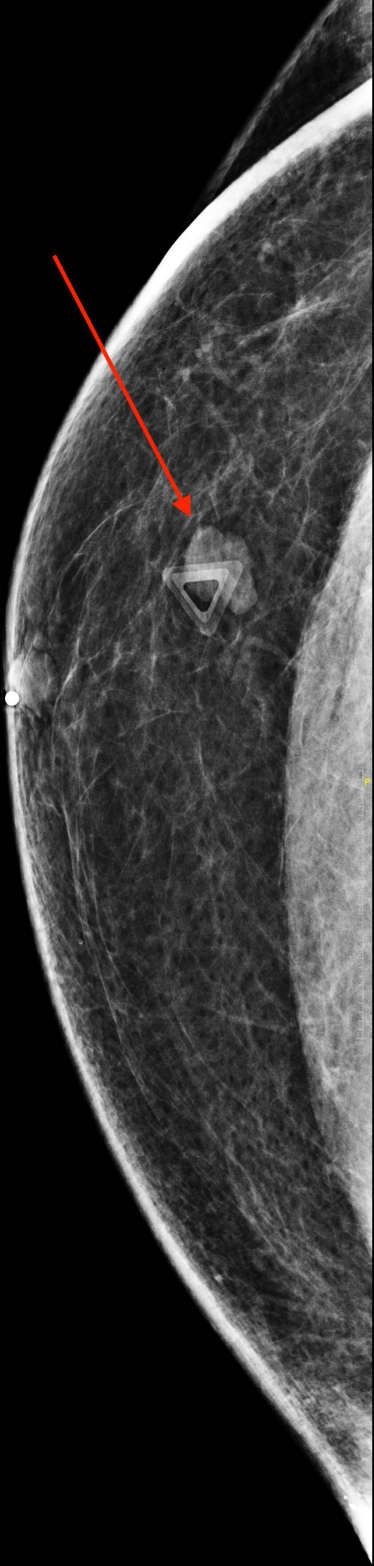
Diagnostic mammogram in the CC projection showing a well-circumscribed mass in the right breast (arrow). CC, craniocaudal.

**Figure 2.**
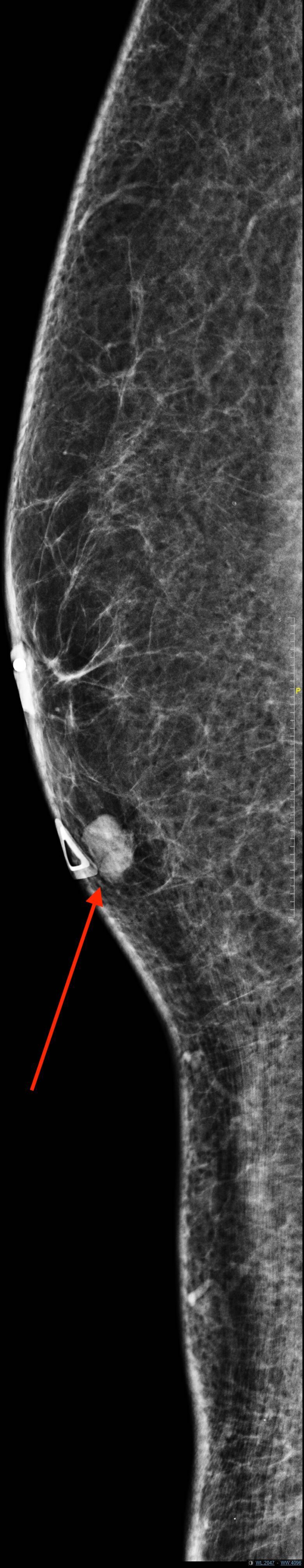
Diagnostic mammogram in the ML projection showing a well-circumscribed mass in the right breast (arrow). ML, mediolateral.

On mammography, a well-circumscribed round mass without associated calcifications or architectural distortion was observed in the right breast (Figures 1 and 2).

**Figure 3.**
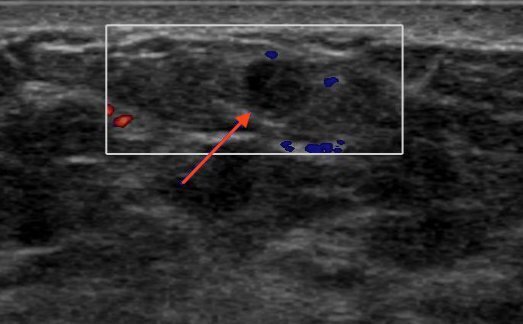
Breast ultrasound confirming the presence of a hypoechoic, well-circumscribed mass on the right side (arrow), with minimal Doppler flow.

A right breast ultrasound showed a well-circumscribed hypoechoic mass with internal septations and echoes. There was minimal associated Doppler flow in the mass (Figure 3). Core needle biopsies of the mass were performed under ultrasound-guidance.

**Figure 4.**
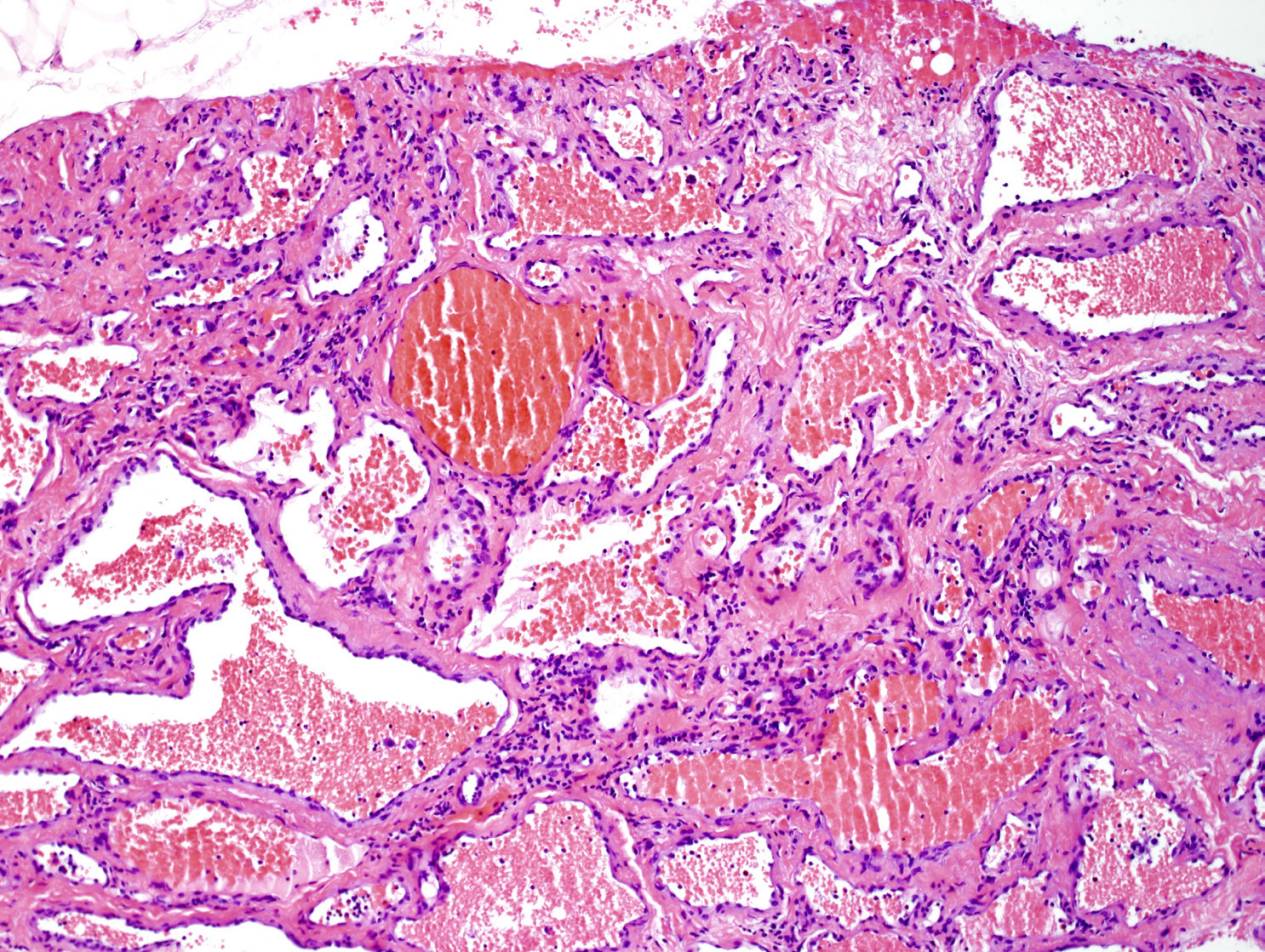
Photomicrograph of the core-needle biopsy showing a hemangioma with multiple dilated blood-filled spaces (H and E, original magnification, x100).

**Figure 5.**
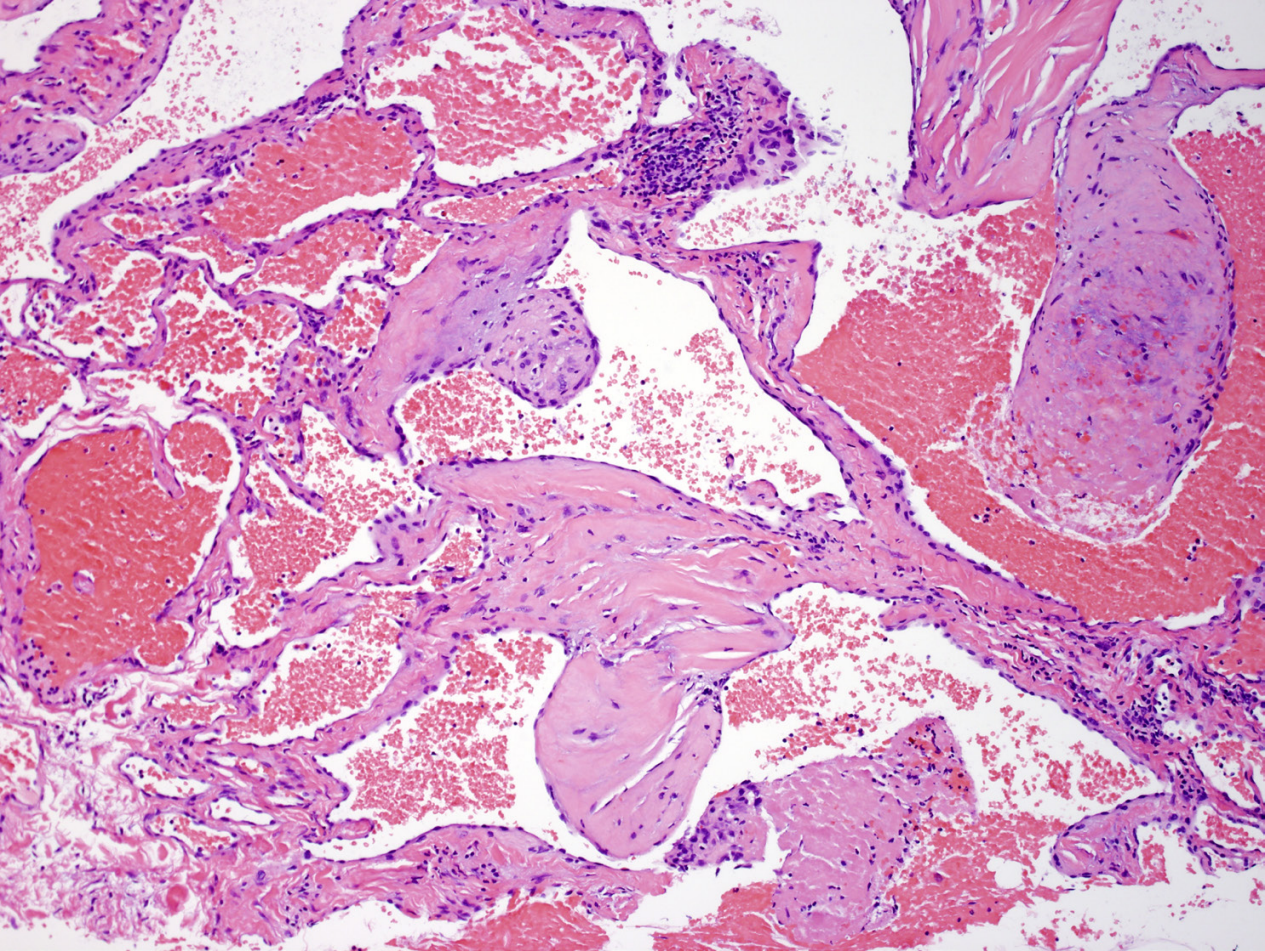
Photomicrograph of the core-needle biopsy of the hemangioma showing an organizing thrombus in a space on the far right and a fibrin thrombus in a vascular space bottom right (H and E, original magnification, x100).

The biopsies showed a hemangioma with predominant features of a cavernous hemangioma. There were variably sized dilated thin-walled vessels, as well as focal feeder muscular veins. No atypia or increased mitotic activity was seen. There were a few thrombosed vascular spaces perhaps related to the recent injury (Figures 4–5).

### Outcome, follow-up, and discussion

The patient was referred for surgical consultation. While some feel that hemangiomas may carry a potential for malignant transformation to angiosarcoma, others feel that “malignant-transformed” lesions were already angiosarcomas to start with, albeit initially underdiagnosed. An example of this is the case reported by Frey et al, where the diagnosis of angiosarcoma was made only after surgical excision of a breast mass initially diagnosed as a hemangioma.^8^

To date, there is no definitive evidence of a cavernous hemangioma undergoing malignant transformation to an angiosarcoma.^9^ A retrospective study by Mesurolle et al, and further highlighted by Yoga et al, concluded that it is reasonable to spare patients surgical excision of benign hemangiomas of the breast with a strong radiographic–pathologic correlation and a clearly benign diagnosis on biopsy.^7,10^ The patient did not undergo surgery and close follow-up was recommended.

Given the possibility that the patient might not have noticed a breast mass in the absence of the preceding thorn prick, it would follow that some breast hemangiomas, along with other benign breast lesions, may be asymptomatic and non-palpable, and thus underreported and underdiagnosed in males.

## Conclusion

Hemangiomas of the breast are rare in males, and almost always present as a palpable mass. However, the case presented here of a patient who might not have been diagnosed with a breast hemangioma without the prior incidental injury suggests that the prevalence of male breast hemangiomas and other benign lesions could be higher than previously thought. Most vascular tumors of the breast are benign, but in some instances, it may be difficult to differentiate a benign vascular tumor from a low-grade malignant vascular tumor in a small biopsy. If the benign characteristics of a hemangioma of the breast are well-supported by biopsy findings and radiographic–pathologic correlation, the patient could be spared surgical resection with close mammographic surveillance and follow-up.

## Learning points

1. Breast masses in males may not always present as a palpable mass, especially in the absence of other symptoms, and may be discovered incidentally.
2. The prevalence of breast masses in males may be underreported due to the asymptomatic nature of some lesions.
3. Vascular tumors of the breast in males are rare, and include both malignant and benign masses, such as angiosarcoma and hemangioma, respectively.
4. Hemangiomas of the breast with a strong radiologic–pathologic correlation and unequivocal benign biopsy findings do not require surgical excision.
5. Mandatory follow-up is necessary if the decision is taken to spare surgery for the patient.
